# Impact of Urbanization and Economic Growth on CO_2_ Emission: A Case of Far East Asian Countries

**DOI:** 10.3390/ijerph17072531

**Published:** 2020-04-07

**Authors:** Asim Anwar, Mustafa Younis, Inayat Ullah

**Affiliations:** 1 Department of Management Sciences, COMSATS University Islamabad, Attock Campus, Attock 43600, Pakistan; Inayat@ciit-attock.edu.pk; 2 Department of Health Policy & Management, Jackson State University, Jackson, MS 39217, USA; younis99@gmail.com

**Keywords:** CO_2_ emission, economic growth, urbanization, fixed effect model, Far East countries

## Abstract

Rising CO_2_ emission constitute a great threat to the world environment and public health. This study examines the major determinants of CO_2_ emissions in Far East countries in the period of 1980 to 2017. We adopt a panel data-fixed effect model that accounts for time-invariant country-specific characteristics that may create omitted-variable bias. We also additionally take care of the time trend by applying an annual fixed effect into our model. The study finds that urbanization, economic growth and trade openness significantly determine CO_2_ emission in the selected countries. Thus, the main policy suggestions are (a) to encourage green and sustainable urbanization, as it helps in economic progress but not at the expense of environmental deterioration; (b) to strategically regulate and improve industrial structure; and (c) enhance sharing of renewable energy in total energy consumption.

## 1. Introduction

Global warming is a major threat to the world environment and public health [1,2]. The massive industrial revolution in recent years ran by huge consumption of fossil fuels and consequent quick increase in emissions of carbon dioxide (CO_2_) has led to austere global warming [3,4,5]. The Intergovernmental Panel on Climate Change (IPCC) reported an increase of average surface temperature by 0.6 °C in the twentieth century ascribed to greenhouse gases [2]. The prevailing global warming and the subsequent climate change pose potential diverse physical, ecological and health threats reciprocated by extreme weather conditions and consequent events like floods, storms, droughts and the heat waves that is accompanied by other fatal conditions like rise in sea level, altered growth of the crops and affected water systems [2,6]. The CO_2_ emission is considered to be a major factor of greenhouse effect and thus apprehend immense attention in recent past among the academics [1].

The IPCC (2014) reveals that CO_2_ emission from industrial processes and fossil fuel combustion shared 78% increase in overall Greenhouse gas emission from 1970 to 2010. The CO_2_ emission since seventies has more than doubled and increased by 40% since 2000. Despite of having stability during 2013 to 2016, the CO_2_ emission have increased by 1.5% in 2017 and continue to increase led by China, India and EU [7]. The lack of policy measures, the emission trends are expected to increase profoundly due to increased global energy demand and fossil fuels being the main drivers of greenhouse gas emissions which will ultimately have negative effects on public health [7].

The empirical literature spanning from last two decades has underscored deep rooted correlation between economic growth and subsequent massive energy usage [8,9]. Energy utilization has been generally recognized as a major factor in relation to CO_2_ emission and in recent years, has been observed as a highly researched topic in different theoretical and empirical investigations. Using STIRPAT model by [10], examined economic growth being an important contributor in promoting carbon dioxide emissions in China. Similar outcomes were originated from the studies conducted in Azerbaijan, Australia and China [11,12,13]. The co-integration technique was adopted to analyze the long run association amid economic development and CO_2_ emission for advanced and less developed nations [14,15,16]. The results indicate output growth, energy consumption and carbon emissions are cross-sectionally dependent and co-integrated. While some researchers explored the causal linkage among economic development, energy consumption and CO_2_ emission applying granger causality technique on both time series and panel dataset are having inconsistent results. A study for India found unidirectional causality from energy consumption to economic growth [17]. While, a study for BRIC countires examined bidirectional causality amid CO_2_ emission and energy usage and bidirectional causality amid energy usage and output growth while unidirectional causalities amid CO_2_ emissions-output growth and energy use and economic development [18]. The studies [1,19,20] did not locate any causality amid CO_2_ emissions and output growth. The researchers have tried to extend the analysis by incorporating trade openness or trade intensity in addition to income/output and financial development [21]. A study using data for newly industrialized countries (NIC) included trade openness into the framework of CO_2_ emissions [22]. The study discovered that trade openness is an important determinant of CO_2_ emissions in NIC. Another study has also evaluated the significance of trade in measuring the level of CO_2_ emission in NIC countries and concluded that trade openness is a critical factor causing emissions [1]. 

Countries in Far East Asia such as Japan, China, South Korea, Malaysia, Singapore, Thailand and Hong Kong have achieved rapid economic growth in last few decades due to division of labor and development of non-agricultural sectors has resulted into an increased urbanization. These countries are among the largest economies of the world with majority of their population lives in urban areas (i.e., 91%, 57.96%, 81.50%, 75.44%, 100%, 49.2% and 100% respectively). These economies with high level of growth and urbanization have contributed enormously to CO_2_ emissions in recent times. Japan, China and South Korea were responsible for 12 percent of the total CO_2_ emissions in 1980 and 18 percent in 2013. Despite having high level of economic growth, urbanization and CO_2_ emission, limited cross country studies have examined the association among urbanization, economic growth and CO_2_ emission in these Far East Asian countries. Therefore, this study aims to study the impact of urbanization and economic development on CO_2_ emissions for the panel of Far East Asian countries. Secondly, we adopt a panel data fixed effect model that accounts for the time-invariant country-specific characteristics that may create omitted variable bias. We also additionally take care of the time trend by applying year fixed effect into our model.

The organizational structure of the article is as follows: Section 2 discusses methodology and data sources of this study; Section 3 offers outcomes and discussion while Section 4 follows with a summary of the results and policy implications.

## 2. Methodology and Variables

Following extensive literature on economic development and energy consumption as the main factors of CO_2_ emission in different parts of the world [10,23], we choose countries with higher carbon emission in Far East Asia. These include China, Japan, South Korea, Malaysia, Singapore, Philippine, Thailand, Hong Kong and Macau. We use the following basic logarithmic transformational model to estimate the effect of covariates on the intensity of CO_2_ emission in the selected region based on the model of [24].

(1) $$\ln{\left(CO2\right)}_{it}=\alpha +\alpha_{1}ln\left(URB_{it}\right)+\alpha_{2}ln\left(GDP_{it}\right)+\alpha_{3}ln\left(TO_{it}\right)+\gamma_{i}+\varepsilon_{it}$$

where the subscript *i* represents the country and *t* shows the time period in years. CO_2_ is the carbon dioxide emission (kt), URB indicates the urbanization (% of total population), GDP specifies the gross domestic product (constant 2010 US$), while TO shows the trade openness. $\gamma_{i}$ represent the individual country-specific time invariant characteristics controlled through fixed effect model. The $\varepsilon_{it}$ is the error term clustered at country level. Data for all these variables was taken from the World Bank Tables.

While using cross-country data, we have number of reasons to use fixed effect model. First, we assume that something within each country may impact or bias the predictor such as GDP, Urbanization or carbon emission, the outcome variables. This potential effect is unobservable; however, we can control for it using fixed effect model. This assumption leads to the possible correlation between individual country’s error term and our explanatory variables. Earlier cross-country studies suggest the use of cluster standard error which is a more conservative yet reliable way of reporting the significance of the estimates. Secondly, we assume that those time-invariant characteristics are unique to the country and may not be correlated with other country’s characteristics. Each country is different and therefore individual country’s error term and the constant (which captures individual country characteristics) may not be correlated with the others. To test this second assumption, we compare the fixed effect estimates with random effect estimates obtained through Equation (2) as follows:

(2) $$\ln{\left(CO2\right)}_{it}=\alpha +\alpha_{1}ln\left(URB_{it}\right)+\alpha_{2}ln\left(GDP_{it}\right)+\alpha_{3}ln\left(TO_{it}\right)+\gamma_{i}+u_{it}+\varepsilon_{it}$$

where $u_{it}$ and $\varepsilon_{it}$ represent cross-country error and within-country error term respectively.

Using Equation (2), the main assumption is country-specific error term is not correlated with the predictors which allows for time-invariant variables to play a role as explanatory variables. In this case, we must specify those variables that may or may not influence the predictors’ variables. Given, that cross-country datasets are always limited to specific variables, therefore, availability of data on all such potential variables is always a limitation. We report the results of both random and fixed effect model along with the differences in the coefficients. Using a long time series, there is always possibility of existence of bias from unobservable factors that change overtime but are fixed to countries. To account for this problem, we employ time fixed effect into our regression by creating year dummies and testing their joint significance through Wald. We failed to reject the null hypothesis that coefficients for all years are jointly equals zero and hence concluded to add year fixed effect into our regression.

## 3. Results and Discussion

The descriptive statistics of the related variables are shown in Table A1. The comparison table reports the results of both the fixed and random effect models along with the difference in the coefficients. The Hausman test shows in Table 1 to use fixed effect model. Our results of panel data fixed effect model are shown in Table 2. In Table 2, column (1) simply tests the effect of urbanization on CO_2_ emission for nine Far East Asian countries without controlling for other covariates. The result shows positive effect of urbanization on CO_2_ emission in these countries. Specifically, CO_2_ emission in studied countries will increase by 2.7 percent with a 1 percent increase in urbanization. However, urbanization is not the only determinant that explains CO_2_ emission. A number of studies [25,26] have used GDP as an important determinant of CO_2_ emission. Hence, we control for GDP in column (2). The coefficient for GDP was statistically significant highlighting economic growth through energy intensity as an important factor in determining CO_2_ emission in the Far East Asian countries. The results in Table 2 verify that 1 percent increase in GDP will lead to increase CO_2_ emission by 0.4 percent in these countries. After controlling GDP, the urbanization effect was still significant at 10% significance level. In addition, in view of the previous studies that suggest trade openness as an important factor contributing into industrial expansion and hence CO_2_ emission, we control for the effect of trade openness in column (3). The results suggest the positive and significant effect of trade openness on CO_2_ emission in the Far East Asian countries. Lastly, as explained in Section 2, we worry about the potential time trend for each country that may affect the covariates over time. Applying year fixed effect in column (4), shows the significance of the responsiveness of carbon emission to urbanization despite controlling all observable factors and country’s fixed effect. More precisely, increasing urbanization by one more percentage is likely to increase carbon emission by nearly 1.5. After controlling for all covariates and country and time year fixed effects, our model captures 85.3% (R^2^) percent variations in the outcome variable that indicates the fitness of the model.

Our results concerning urbanization are in line with other studies. For instance, [13] has termed urbanization as positive contributor to CO_2_ emission in China. Likewise, a study for Pakistan found a positive effect of urbanization on CO_2_ emission using ARDL and VECM models [27]. Urbanization contributes to sustainable economic growth by increasing productivity and innovation [28]. The reports suggest that 50% of the global population lives in metropolises and is increasing daily, while 80% of the worldwide GDP produced in metropolises. However, the increased urbanization brings challenges as they consume two-thirds of the total energy and contribute 70% of total greenhouse gas emissions [28]. The modern ecological and environmental theories, researchers highlighted the presence of inverted U-shaped association amid pollution and urbanization, and therefore with increased urbanization, the relationship may change from positive to negative. Urbanization in Far East Asian countries is surging in the recent past, making the environment more polluted than other countries. Increased urbanization in these countries shows an increase in economic output per capita in these economies. These countries are paying higher attention to counter the negative effects of urbanization which they are achieving by lowering emissions and more usage of renewable energy. As the same time, higher levels of urbanization has serious effects on CO_2_ emissions in these countries having serious consequences for overall population health

Our results also indicate the positive and significant impact of economic growth on CO_2_ emission, which corroborates with the previous studies [25,26]. Researchers have found significant associations between GDP and CO_2_ emission in transitional economies [25]. Researchers have also found positive effect of economic growth and CO_2_ emission in developed, emerging and middle & north African countries [26]. The phenomenal economic growth in the studied countries in last few decades due to industrial and manufacturing booms has increased the demand for energy consumption which results in an increase in CO_2_ emissions [4,5,22]. These countries were responsible for almost 18% of total global CO_2_ emissions in 2013 from petroleum consumption. The IPCC, in its report, highlighted that fossil fuel combustion and cement manufacturing are responsible for 75% of human-caused CO_2_ emissions. Industrialization increases income per capita, and the opportunities provided with increases in income further enhances the demand for energy at individual—as well as at the sector level—which result in an increases in CO_2_ emissions. The thrust for achieving higher and sustainable economic growth using energy resources has outpaced the positive externalities to negative in terms of environmental degradation in Far East Asian countries. China, Japan and South Korea has introduced and implemented various schemes for tackling air pollution in East Asia. These schemes include Long-Range transboundary Pollution of China, South Korea and Japan (LTP), Acid Deposition Monitoring Network in East Asia (EANET) and North-East Asian Subregional Program for Environmental Cooperation (NEASPEC). These schemes unfortunately underperformed in creating an effective international regime in controlling transboundary air pollution in Far East Asian region. The increase in CO_2_ emission has increased the number of deaths and other health related problems like respiratory diseases, heart problems and stroke in these countries. Most of the Far East countries have started programs to reduce carbon emissions through decrease in energy consumption by increasing electrification and promoting capacity for clean oil, coal, nuclear and renewable energies.

The positive effect of trade on CO_2_ emission in Far East Asian countries suggests an increase in the amount of trade will tend to increase the level of CO_2_ emission. The result of this study is in line with [29] which shows a positive relationship between trade openness and CO_2_ emission in 105 high, middle- and low-income countries. Everything remaining constant, increase in trade will lead to increased energy usage, which is an important factor in rising CO_2_ emission. Despite of having positive externalities of trade it has some negative externalities in terms of environmental degradation [22]. The production of more exportable goods and transportation of exportable and importable is attributed to more CO_2_ emission through combustion of more fossil fuels, oil spills and emission of Sulfur and carbon [30]. This indicates that domestic and foreign investors in Far East countries do not utilize environment friendly technologies in production processes which create health related issues for the masses.

## 4. Conclusions 

Over the last few decades, the Far East Asian countries have achieved remarkable economic growth. However, along with such spectacular growth, East Asian countries face the challenge of increasing CO_2_ emissions.

The present study examined the relationship among CO_2_ emissions (kt), economic growth (GDP), urbanization (% of total population) and trade openness for a panel of nine Far East Asian countries from 1980 to 2017, using a fixed effect approach.

Our estimates show that GDP, urbanization and trade openness were significantly correlated with CO_2_ emission in the panel of Far East Asian countries. Far East Asian countries are growing at a fast rate in terms of GDP, levels of urbanization and CO_2_ emissions, according to the World Bank collection of development indicators. This fast track to increase in GDP, urbanization and trade has caused environmental degradation, causing serious health issues in these countries.

### Policy Implications

Energy demand is expected to grow globally, but especially in East Asia, where China is the largest energy consumer with 23 percent of the global consumption. The total primary energy consumption in the studied countries has risen by 21 percent, while power consumption has increased by 43 percent during 2005 to 2015. Considering the pace of economic growth and urbanization in Far East Asian countries, the energy consumption will double by 2040, where conventional energy may still be used to meet growing energy demand. Therefore, considering the growing demand for energy, the findings of this study have important policy implications for Far East countries discussed below.

Far East countries should design more energy conservation policies to limit CO_2_ emission. The studied countries need to promote and facilitate green and sustainable urbanization to improve and sustain economic progress without any ecological mortification. Governments need to promote renewable energy consumption in urban areas to strengthen the structure of energy usage so as to enhance the effect of renewable energy on subsequent urbanization such as heating system, solar lighting and ethane gasoline for vehicles. Any improvement in energy efficacy and promoting high-tech innovation can help in reducing energy intensity. The lower energy usage will help in reducing CO_2_ emission in Far East countries which may help in improving the health standard in these countries. Thus, these governments should further promote R&D to improve the non-renewable energy efficiency and decrease renewable energy costs.

The chemical and heavy industries being the key contributors in CO_2_ emission play a vital role in the Far East Asian countries industrial growth. Therefore, these countries need to deepen the reform of these industries through administrative means and encouraging zero and light emission industry through promoting industrial diversity. Furthermore, businesses and individuals should decrease energy waster and encourage recycling to increase their environmentally friendly consciousness. Better and timely policies designed to reduce CO_2_ emission will improve the health conditions of urban population, as well as populations at large.

## Figures and Tables

**Table 1:** Comparison of fixed and random effect models.

	(1)	(2)	(3)
Dep. Var: Log of CO_2_ Emission	Fixed Effect Model	Random Effect Model	Difference (Fixed−Random)
Log of Urbanization	1.230 ***	0.617 ***	0.613 ***
	(0.161)	(0.191)	(0.0789)
Log of GDP	0.250 ***	0.573 ***	−0.323 ***
	(0.0530)	(0.0552)	(0.039)
Log of Trade Openness	0.176 ***	−0.0239	0.199 ***
	(0.0598)	(0.0681)	(0.035)
Constant	−0.441	−6.029 ***	
	(1.261)	(1.383)	
Observations (N)	313	313	313
R−squared	0.803		
Hausman Test (Prob > chi2)			0.0000
Number of country1	9	9	9

Robust standard errors in parentheses *** *p* < 0.01, * *p* < 0.1.

**Table 2:** Main determinants of carbon emission (CO_2_) in Far-East Asian countries (fixed effect model).

Dep. Var: CO_2_ Emission	(1)	(2)	(3)	(4)
Log of Urbanization	2.718 ***	1.370 *	1.149 *	1.426 **
	(0.539)	(0.622)	(0.605)	(0.596)
Log of GDP		0.410 **	0.237 **	0.0222
		(0.123)	(0.0959)	(0.244)
Log of Trade Openness			0.303 *	0.122
			(0.139)	(0.212)
Country FE	YES	YES	YES	YES
Year FE (Time Trend)	NO	NO	NO	YES
Constant	0.280	−4.821	0.751	3.717
	(2.237)	(2.714)	(3.373)	(5.275)
Observations	315	313	313	313
R-squared	0.656	0.775	0.800	0.853
Number of country1	9	9	9	9

Note: Table 2 shows the panel fixed effect estimates of the CO_2_ emission in nine Far East Asian countries using a balanced panel data from 1980 to 2017. Standard errors are clustered at country level shown in parenthesis. Dependent variable is the carbon dioxide emission measured in metric tons. *** *p* < 0.01, ** *p* < 0.05, * *p* < 0.1.

## Appendix A

**Table A1:** Descriptive Statistics.

	Count	Mean	Sd	Min	Max
CO_2_ Emission (Metric Tons)	315	701,051.8	1,642,323	484.044	1.03 × 10^7^
GDP (constant at USD$)	340	1.08 × 10^12^	1.97 × 10^12^	4.13 × 10^9^	1.02 × 10^13^
Urbanization (% of total population)	342	70.17376	26.51184	19.358	100
Trade Openness	340	1.091394	1.120074	0.0695748	8.447878
Observations	342				

Note: CO_2_ is the carbon dioxide emission measured in (kt), URB indicates the urbanization (% of total population), GDP specifies the gross domestic product (constant 2010 US$), while TO shows trade openness.
